# Allergy diagnostic performance of FastCheckPOC 20 Atopy

**DOI:** 10.3389/falgy.2025.1669268

**Published:** 2025-10-24

**Authors:** Hannes Nösslinger, Ewald Mair, Gertie J. Oostingh, Verena Ahlgrimm-Siess, Anna Ringauf, Roland Lang

**Affiliations:** 1 Department of Nutrition and Dietetics, Hospital of Merano (SABES-ASDAA), Teaching Hospital of Paracelsus Medical University, Merano-Meran, Italy; 2 Registered Pediatrician (SABES-ASDAA), Brunico-Bruneck, Italy; 3 Biomedical Sciences, Salzburg University of Applied Sciences, Puch/Salzburg, Austria; 4 Department of Dermatology and Allergology, University Hospital of the Paracelsus Medical University, Salzburg, Austria; 5 MacroArray Diagnostics (MADx), Vienna, Austria

**Keywords:** allergy and immunology, atopy, IgE, lateral flow test, molecular diagnostics, point-of-care test, primary care

## Abstract

**Background:**

The increasing prevalence of allergic diseases, along with their diagnosis and treatment, presents a growing challenge in health care. To reduce this burden, a highly sensitive and specific point-of-care test for detecting sensitization could be implemented in a primary health care setting. The study aimed to investigate the accuracy of FastCheckPOC 20 Atopy (FCP20) in comparison with the multiplex assay Allergy Explorer 2 (ALEX^2^) system.

**Methods:**

In this cross-sectional study, 215 participants were recruited from South Tyrol, Italy. Serum samples were analyzed using both FCP20 and ALEX^2^. Dichotomous data were used to calculate sensitivity and specificity in comparison with the ALEX^2^.

**Results:**

The overall sensitivity of the FCP20 was 43.3% (95% CI: 40.3%–46.2%), and the specificity was 92.1% (95% CI: 91.1%–93.0%). Inhalation allergens showed a higher sensitivity than food allergens; the grass pollen (gx17) exhibited the highest sensitivity at 79.8% (95% CI: 72.6%–85.7%). Among patients with severe allergic symptoms, bronchial asthma, or eczema, sensitivity increased to over 83%.

**Conclusions:**

FCP20 demonstrates high specificity and may be considered for the exclusion of sensitization to selected allergens, but its low sensitivity limits its utility as a general screening tool.

## 1 Introduction

The prevalence of allergic diseases, which are a burden for affected patients and their families, is increasing. Furthermore, they impose an economic burden on society if they are not properly diagnosed and treated (1, 2). Unfortunately, a high number of affected patients have been identified as undiagnosed and not receiving satisfactory treatment (2).

Most patients presenting with allergies are first seen in the primary care setting, which includes general and family practitioners as well as pediatricians (3, 4). Since the diagnosis and management of allergic diseases in primary care are not sufficiently established, strategies, educational opportunities, and tools to support primary healthcare providers need to be developed (4, 5). Point-of-care (POC) testing based on lateral flow technology has been designed for primary care providers and has been applied in the field of allergology since the late 1990s (6). The practical utility of POC devices for diagnostic evaluation depends on their accuracy. The performance of POC devices for the detection of specific IgE has mainly been evaluated by comparing the ImmunoCAP Rapid (Thermo Fisher Scientific, Uppsala, Sweden) with the ImmunoCAP (Thermo Fisher Scientific, Uppsala, Sweden) (7–11).

The FastCheckPOC 20 Atopy (FCP20) (DST Diagnostische Systeme & Technologien GmbH, Schwerin, Germany) is another POC device used in the field of allergy diagnostics. To the best of our knowledge, except for Babakhin et al. (12) and Kamath et al. (13), who presented two poster studies, Rübenhagen et al. (14) published the only study to date that examined the performance of the FCP20. Compared with the ImmunoCAP, Rübenhagen et al. found a sensitivity and specificity over all 20 allergens of 76% and 80%, respectively. Compared with the skin prick test, they found a sensitivity and specificity for all 20 allergens combined of 66% and 71%, respectively (14).

This study aimed to evaluate the performance of the FCP20 in comparison with the multiplex assay Allergy Explorer 2 (ALEX^2^) [MacroArray Diagnostics (MADx), Vienna, Austria].

## 2 Materials and methods

### 2.1 Research design and ethical approval

This was a prospective, observational, descriptive, monocentric, and cross-sectional study. The study was approved by the Ethics Committee of the South Tyrolean Health Authority (number: 136–2020), and the protocols of the Declaration of Helsinki were adhered to. Written informed consent was obtained from all participants. For participants below 18 years of age, their legal representatives provided informed consent to participate in this study.

### 2.2 Recruitment and sample collection

A total of 215 patients were recruited from the Department of Nutrition and Dietetics, Hospital of Merano, and a pediatric surgery in Brunico, South Tyrol, Italy. The inclusion criteria encompassed positive sensitizations to any food or aeroallergen, as determined by skin prick tests previously performed or by the presence of sIgE levels ≥0.30 kU_A_/L and/or allergic symptoms to any food and/or aeroallergen. Of the 215 subjects, 14 (6.5%) demonstrated no sensitization in ALEX^2^, which was used as the negative control in our analysis. Of these 14 subjects, only one did not present allergic or allergic-like symptoms. The recruitment and examination period, including blood extraction for serum collection and the FCP20 and ALEX^2^ tests, took place from 1 February 2021 to 5 November 2021. Participants' allergy history was obtained at the beginning of each examination. An allergy history questionnaire (QUETHEB allergy questionnaire from the German Society of Qualified Nutritional Therapists and Nutritionists) (15) was used to aid in standardizing symptom description.

Venous blood was collected from each participant, and the sample was left undisturbed for 15–30 min to allow blood clotting. Thereafter, the serum was obtained after centrifugation at 1,500 × *g* for 10 min in a refrigerated centrifuge, and 2 mL of serum was transferred into each of two new tubes (one for the FCP20 test and one for the ALEX^2^ test). The tubes for the ALEX^2^ tests were stored at −20°C. These frozen samples were transported in a portable EVERmed refrigerator PR11 at −18°C to MADx, Vienna, where the ALEX^2^ tests were performed according to the manufacturer's instructions. The serum from the tubes for the FCP20 test was used immediately to perform the FCP20 test. Each blood sample tube and the ALEX^2^ microchip were carefully checked visually before measurement to exclude potential sources of error.

### 2.3 FCP20 tests

The FCP20 is a POC test based on lateral flow technology. The cassette measures approximately 9.5 cm × 5 cm × 0.7 cm. It is an *in vitro* semi-quantitative enzyme immunoassay for the parallel measurement of allergen-specific human IgE to 20 allergens and allergen mixtures (Table 1) in heparinized or Na-EDTA, venous or capillary blood, plasma or serum. After collecting the blood sample, it is diluted with a sample diluent. Thereafter, three different washing solutions and a buffer solution are injected. It takes 30 min to perform these steps until the results are ready. The results are obtained by optical evaluation and can be classified into five levels with a correlation to carrier–polymer system (CAP) classes (16). CAP class 2 is usually seen as a borderline sensitization (16); therefore, FCP20 levels 2–5 were classified as positive, and FCP20 level 1 was classified as negative.

**Table 1:** Comparison of FCP20 extracts with the sum of corresponding extracts and components in ALEX^2^.

Allergen species	FCP20 extracts	ALEX^2^ extracts	ALEX^2^ components
*Ambrosia artemisiifolia* ^a^	w1	Amb a	Amb a 1, Amb a 4
*Artemisia vulgaris* ^a^	w6	Art v	Art v 1, Art v 3
*Phleum pratense* ^a^	g6		Phl p 1, Phl p 2, Phl p 5.0101, Phl p 6, Phl p 7, Phl p 12
^c^	gx17		Lol p 1
*Secale cereale* ^a^	g12	Sec c pollen	
*Betula verrucosa* ^a^	t3		Bet v 1, Bet v 2, Bet v 6
*Ficus benjamina*^a^, *Hevea brasiliensis*^a^	kx1	Fic b	Hev b 1, Hev b 3, Hev b 5, Hev b 6.02, Hev b 8, Hev b 11
*Felis domesticus* ^a^	e1		Fel d 1, Fel d 2, Fel d 4, Fel d 7
*Canis familiaris* ^a^	e5	Can f male urine	Can f 1, Can f 2, Can f 3, Can f 4, Can f 6, Can f_Fd1
*Dermatophagoides farinae*^a^, *Dermatophagoides pteronyssinus*^a^	dx2		Der f 1, Der f 2, Der p 1, Der p 2, Der p 5, Der p 7, Der p 10, Der p 11, Der p 20, Der p 21, Der p 23
*Ovum gallinae* ^b^	f1	Gal d white	Gal d 1, Gal d 2, Gal d 3, Gal d 4
*Bos primigenius taurus* ^b^	f199	Bos d milk	Bos d 4, Bos d 5, Bos d 6, Bos d 8
*Gadus morhua* ^b^	f3	Gad m	Gad m 1, Gad m 2 + 3
*Triticum aestivum* ^b^	f4		Tri a 14, Tri a 19, Tri a aA_TI
*Arachis hypogaea* ^b^	f13		Ara h 1, Ara h 2, Ara h 3, Ara h 6, Ara h 8, Ara h 9, Ara h 15
*Glycine max* ^b^	f14		Gly m 4, Gly m 5, Gly m 6, Gly m 8
*Apium graveolens*	f85		Api g 1, Api g 2, Api g 6
*Prunus amygdalus* ^b^	f20	Pru du	
*Juglans regia* ^b^	f16		Jug r 1, Jug r 2, Jug r 3, Jug r 4, Jug r 6
*Corylus avellana* ^b^	f17		Cor a 1.0401, Cor a 8, Cor a 9, Cor a 11, Cor a 14

a Inhalant allergen.

b Food allergen.

c *Anthoxanthum odoratum*^a^, *Dactylis glomerata*^a^, *Lolium perenne*^a^, *Poa pratensis*^a^.

The FCP20 tests performed in our study were carried out according to the manufacturer's instructions. The test results were categorized as positive and negative (if the intensity of the signal band was less than the lower standard). The tests were always carried out and analyzed by the same person under constant lighting conditions to avoid a possible bias (17).

### 2.4 Specific IgE testing using the multiplex assay ALEX^2^

ALEX^2^ analyzes IgE reactivity against 295 allergens from 165 allergen sources in a single blood sample. Compared with other multiplex tests, the novelties of the ALEX^2^ microchip include the addition of 117 allergen extracts to 178 molecular allergen components and an inhibitor that suppresses the binding of IgE to clinically irrelevant, cross-reacting carbohydrate determinants (CCDs) (17).

ALEX^2^ testing in our study was performed according to the manufacturer's instructions. Test results were expressed quantitatively in kilounits of allergen-specific IgE per liter (kU_A_/L) and categorized into five specific IgE (sIgE) classes: negative or uncertain (<0.3 kU_A_/L), low (0.3 to <1 kU_A_/L), moderate (1 to <5 kU_A_/L), high (5 to <15 kU_A_/L), and very high (≥15 kU_A_/L) (17).

### 2.5 Symptoms

Respiratory (rhinitis, conjunctivitis, bronchitis, and bronchial asthma), gastrointestinal (oral allergy syndrome, vomiting, and diarrhea), and cutaneous (eczema, itching, and urticaria) symptoms were assessed. Acute allergic symptoms were assessed by the same physician during the clinical examination. Past or intermittent symptoms were recorded during the interview with the aid of the QUETHEB allergy history questionnaire (15) to standardize symptom description. Symptom intensity was subjectively reported by patients and classified as low, low to moderate, moderate, moderate to high, and high. Moderate to high and high symptom intensities were categorized as severe allergic symptoms. The diagnosis of bronchial asthma was supported by fractional exhaled nitric oxide (FeNO) measurements, and eczema was classified according to the Investigator Global Assessment Score (IGA score).

### 2.6 Statistics

Qualitative results of the FCP20 were compared with quantitative data from the ALEX^2^. FCP20 allergens and corresponding ALEX^2^ extracts and components are listed in Table 1. All 20 allergens of FCP20 were classified as positive (FCP20 levels 2–5) or negative (FCP20 level 1) and compared with the sum of quantitative sIgE of corresponding extracts and components in ALEX^2^ and not with individual components alone. A positive FCP20 test and at least one positive corresponding extract or component in ALEX^2^ (sIgE ≥ 0.3 kU_A_/L) were considered as a positive agreement. A negative agreement existed if the FCP20 test and the corresponding extracts or components in ALEX^2^ were negative.

For all 20 allergens in FCP20, sensitivity, specificity, area under the curve (AUC), positive and negative likelihood ratios, positive and negative predictive value (PPV and NPV), and accuracy were calculated with 95% CI.

Sensitivity of FCP20 was also calculated using the following classification of sensitization in ALEX^2^: 0.30–0.99 kU_A_/L (class 1), 1.00–4.99 kU_A_/L (class 2), 5.00–14.99 kU_A_/L (class 3), and ≥15 kU_A_/L (class 4).

Data were analyzed using MedCalc® Statistical Software Version 22.013 (MedCalc Software Ltd., Ostend, Belgium; https://www.medcalc.org; 2023).

## 3 Results

A total of 215 test subjects with existing allergies or suspected allergy symptoms were analyzed, including 122 males and 93 females (56.7% vs. 43.3%). Of these, 116 patients were <18 years old (54.0%), and 99 patients (46.0%) were >18 years old, resulting in a median age of 15 years (range 1–76). Compared with the ALEX^2^ system as the reference method, the overall sensitivity of the FCP20 was 43.3% (95% CI: 40.3%–46.2%), and the overall specificity was 92.1% (95% CI: 91.1%–93.0%). The PPV was 66.0% (95% CI: 62.0%–69.0%), and the NPV was 81.9% (95% CI: 81.2%–82.7%). The accuracy was 79.2% (95% CI: 78.0%–80.4%) when compared with the ALEX^2^ system.

For inhalant allergens, the FCP20 test resulted in a higher sensitivity [46.3% (95% CI: 43.0%–49.6%)] and higher specificity [95.6% (95% CI: 94.3%–96.6%)] (Table 2) in comparison with food allergens [32.0% (95% CI: 26.1%–38.2%) and 89.7% (95% CI: 88.3%–91.1%)], respectively (Table 3). Whereas the PPV for inhalant allergens was substantially higher in comparison with food allergens [88.1% (95% CI: 85.0%–90.6%) vs. 28.2% (95% CI: 23.8%–33.0%)], the NPV for inhalant allergens [71.5% (95% CI: 70.2%–72.8%)] was lower when compared with food allergens [91.3% (95% CI: 90.5%–92.0%)].

**Table 2:** Performance of FCP20 inhalant allergen extracts as compared with the sum of corresponding ALEX^2^ extracts and components.

FCP20	Sensitivity (%)	Specificity (%)	AUC	Prevalence (%)	PPV (%)	NPV (%)	Accuracy (%)
w1	42.9 (17.7–71.1)	89.1 (83.9–93.0)	0.66 (0.59–0.72)	6.5 (3.6–10.9)	21.4 (11.7–36.0)	95.7 (93.4–97.3)	86.1 (80.7–90.4)
w6	24.3 (11.8–41.2)	62.8 (56.7–68.6)	0.44 (0.38–0.49)	12.2 (8.8–16.4)	8.3 (4.8–14.1)	85.6 (82.9–88.0)	58.1 (52.3–63.7)
g6	71.7 (64.3–78.3)	97.6 (87.4–99.9)	0.85 (0.79–0.89)	80.5 (74.5–85.5)	99.2 (94.7–99.9)	45.6 (39.7–51.6)	76.7 (70.5–82.2)
gx17	79.8 (72.6–85.7)	93.1 (83.0–98.1)	0.86 (0.81–0.91)	73.5 (67.1–79.3)	96.9 (92.4–98.8)	62.4 (54.7–69.5)	83.3 (77.6–88.0)
g12	63.2 (54.8–71.1)	93.0 (84.3–97.7)	0.78 (0.72–0.83)	67.0 (60.3–73.2)	94.8 (88.6–97.7)	55.5 (49.9–60.9)	73.0 (66.6–78.8)
t3	10.6 (5.6–17.8)	97.1 (91.7–99.4)	0.54 (0.47–0.61)	52.6 (45.7–59.4)	80.0 (53.7–93.2)	49.5 (47.7–51.3)	51.6 (44.7–58.5)
kx1	10.7 (2.3–28.2)	97.3 (93.9–99.1)	0.54 (0.47–0.61)	13.0 (8.8–18.3)	37.5 (13.2–70.4)	87.9 (86.5–89.2)	86.1 (80.7–90.4)
e1	15.0 (8.7–23.5)	100 (96.8–100)	0.58 (0.51–0.64)	46.5 (39.7–53.4)	100 (78.2–100)	57.5 (55.5–59.5)	60.5 (53.6–67.1)
e5	23.7 (13.6–36.6)	98.1 (94.5–99.6)	0.61 (0.54–0.68)	27.4 (21.6–33.9)	82.4 (58.2–94.0)	77.3 (74.7–79.7)	77.7 (71.5–83.1)
dx2	19.7 (10.9–31.3)	98.7 (95.2–99.8)	0.59 (0.52–0.66)	30.7 (24.6–37.3)	86.7 (60.1–96.6)	73.5 (71.1–75.8)	74.4 (68.0–80.1)

AUC, area under the curve; prevalence, sensitization prevalence in ALEX^2^; PPV, positive predictive value; NPV, negative predictive value. The 95% CI is shown in brackets.

**Table 3:** Performance of FCP20 food allergen extracts as compared with the sum of corresponding ALEX^2^ extracts and components.

FCP20	Sensitivity (%)	Specificity (%)	AUC	Prevalence (%)	PPV (%)	NPV (%)	Accuracy (%)
f1	36.4 (10.0–69.2)	100 (98.2–100)	0.68 (0.62–0.74)	5.1 (2.6–9.0)	100 (39.8–100)	96.7 (94.9–97.9)	96.7 (93.4–98.7)
f199	33.3 (4.3–77.7)	82.5 (76.7–87.3)	0.58 (0.51–0.65)	2.8 (1.0–5.9)	5.1 (1.7–14.8)	97.8 (96.1–98.7)	81.1 (75.3–86.1)
f3	100 (15.8–100)	96.2 (92.7–98.4)	0.98 (0.95–1.00)	0.93 (0.1–3.3)	20.0 (11.2–33.0)	100 (98.2–100)	96.3 (92.8–98.4)
f4	66.7 (9.4–99.2)	90.6 (85.8–94.1)	0.79 (0.73–0.84)	1.4 (0.3–4.0)	9.1 (3.9–19.8)	99.5 (97.5–99.9)	90.2 (85.5–93.9)
f13	15.4 (5.9–30.5)	100 (97.9–100)	0.58 (0.51–0.64)	18.1 (13.2–24.0)	100 (54.1–100)	84.2 (82.4–85.9)	84.7 (82.4–85.9)
f14	30.3 (15.6–48.7)	90.1 (84.8–94.0)	0.60 (0.53–0.67)	15.4 (10.8–20.9)	35.7 (22.0–52.3)	87.7 (85.0–90.0)	80.9 (75.0–86.0)
f85	46.3 (30.7–62.6)	67.2 (59.7–74.2)	0.57 (0.50–0.64)	19.1 (14.1–25.0)	25.0 (18.4–33.1)	84.2 (79.7–87.8)	63.3 (56.4–69.7)
f20	42.9 (9.9–81.6)	94.2 (90.1–97.0)	0.69 (0.62–0.75)	3.3 (1.3–6.6)	20.0 (8.3–40.9)	98.0 (96.3–98.9)	92.6 (88.1–95.7)
f16	75.0 (47.6–92.7)	84.9 (79.2–89.6)	0.80 (0.74–0.85)	7.4 (4.3–11.8)	28.6 (20.6–38.2)	97.7 (94.8–99.0)	84.2 (78.6–88.8)
f17	20.5 (12.4–30.8)	89.4 (82.9–94.1)	0.55 (0.48–0.62)	38.6 (32.1–45.5)	54.8 (38.8–70.0)	64.1 (61.2–66.9)	62.8 (56.0–69.3)

AUC, area under the curve; prevalence, sensitization prevalence in ALEX^2^; PPV, positive predictive value; NPV, negative predictive value. The 95% CI is shown in brackets.

Large differences in the evaluated parameters were observed between the individual FCP20 allergens (Tables 2, 3). Among inhalant allergens, the sensitivity ranged from 10.6% (95% CI: 5.6%–17.8%) for *Betula verrucosa* (t3) to 79.8% (95% CI: 72.6%–85.7%) for a mix of *Anthoxanthum odoratum*, *Dactylis glomerata*, *Lolium perenne*, and *Poa pratensis* (gx17), whereas specificity was generally higher ranging from 62.8% (95% CI: 56.7%–68.6%) for *Artemisia vulgaris* (w6) to 100% (95% CI: 96.8%–100%) for *Felis domesticus* (e1). An acceptable (or higher) performance of individual inhalant allergens as determined by AUC was only observed for *Secale cereale* (g12) with an AUC of 0.78 (95% CI: 0.72–0.83), *Phleum pratense* (g6) [AUC 0.85 (95% CI: 0.79–0.89)], and a mix of *Anthoxanthum odoratum*, *Dactylis glomerata*, *Lolium perenne*, and *Poa pratensis* (gx17) [AUC 0.86 (95% CI: 0.81–0.9)]. The PPV was lowest for *Artemisia vulgaris* (w6) [8.3% (95% CI: 4.8%–14.1%)] and *Ambrosia artemisiifolia* (w1) [21.4% (95% CI: 11.7%–36%)], whereas the PPV was highest for *Phleum pratense* (g6) and *Felis domesticus* (e1) [99.2% (95% CI: 94.7%–99.9%) and 100% (95% CI: 78.2%–100%), respectively]. The NPV ranged from 45.6% (95% CI: 39.7%–51.6%) for *Phleum pratense* (g6) to 95.7% (95% CI: 93.4%–97.3%) for *Ambrosia artemisiifolia* (w1).

Among food allergens, the sensitivity ranged from 15.4% (95% CI: 5.9%–30.5%) for *Arachis hypogaea* (f13) to 100% (95% CI: 15.8%–100%) for *Gadus morhua* (f3), whereas specificity was generally higher ranging from 67.2% (95% CI: 59.7%–74.2%) for *Apium graveolens* (f85) to 100% (95% CI: 98.2%–100%) for *Ovum gallinae* (f1) and 100% (95% CI: 97.9%–100%) for *Arachis hypogaea* (f13). An acceptable (or higher) performance of individual food allergens as determined by AUC was only observed for *Triticum aestivum* (f4) with an AUC of 0.79 (95% CI: 0.73–0.84), *Juglans regia* (f16) [AUC 0.80 (95% CI: 0.74–0.85)], and *Gadus morhua* (f3) [AUC 0.98 (95% CI: 0.95–1.00)]. The PPV was lowest for *Bos primigenius taurus* (f199) (5.1% (95% CI: 1.7%–14.8%), whereas the PPV was highest for *Ovum gallinae* (f1) and *Arachis hypogaea* (f13) [100% (95% CI: 39.8%–100%) and 100% (95% CI: 54.1%–100%), respectively]. The NPV ranged from 64.1% (95% CI: 61.2%–66.9%) for *Corylus avellana* (f17) to 100% (95% CI: 98.2%–100%) for *Gadus morhua* (f3).

ROC curves were constructed for each individual allergen component and extract in ALEX^2^ (Supplementary Figures S1 and S2) to evaluate the concordance between FCP20 and ALEX^2^. These curves identify the components or extracts that demonstrate the strongest agreement between the two diagnostic tests.

**Figure 1:**
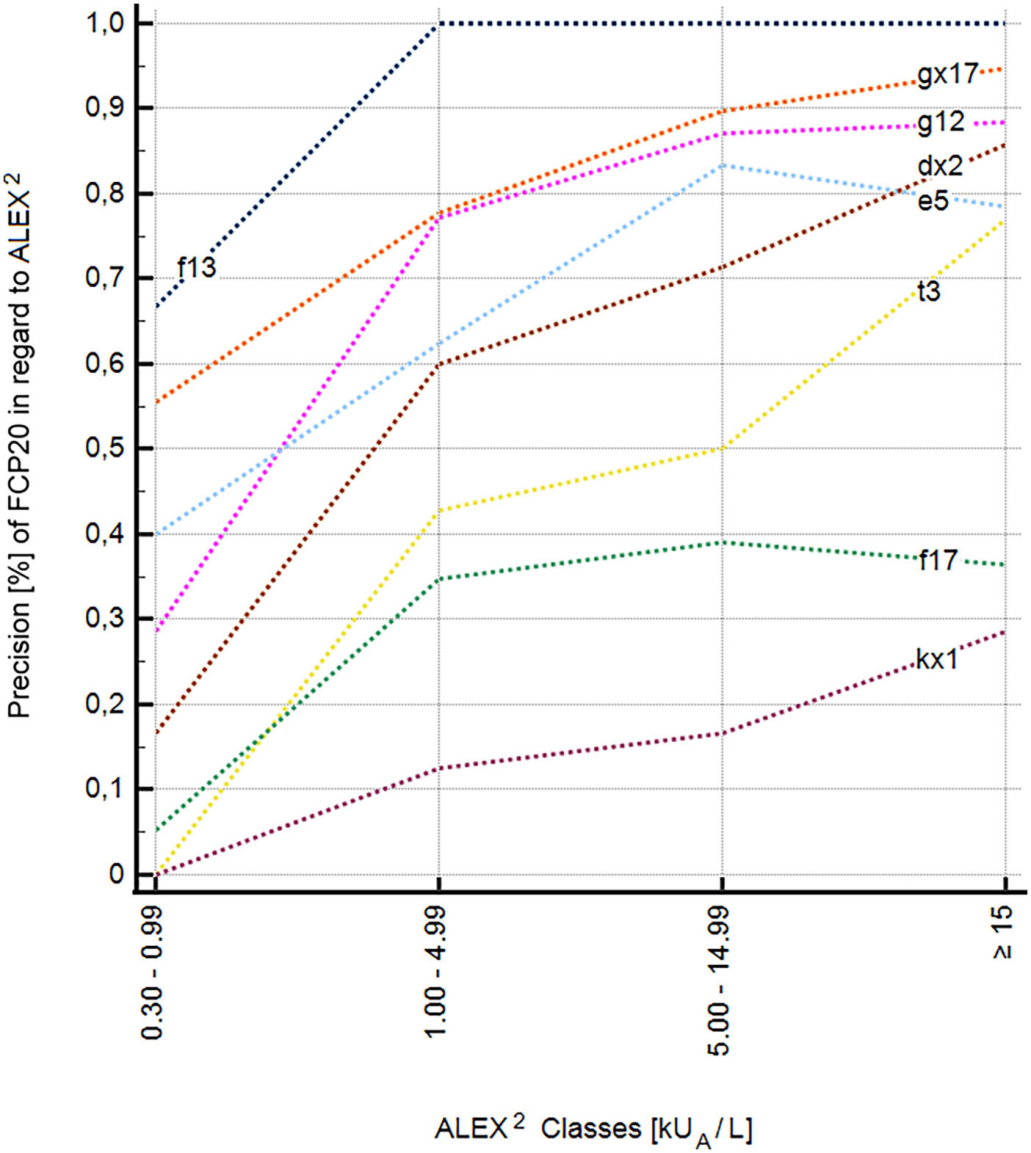
Increase in precision of eight individual allergens of the FCP20 with increasing specific IgE values to extracts and components in ALEX^2^. dx2, *Dermatophagoides farinae*, *Dermatophagoides pteronyssinus*; e5, *Canis familiaris*; f13, *Arachis hypogaea*; f17, *Corylus avellana*; g12, *Secale cereale*; gx17, *Lolium perenne*; kx1, *Ficus benjamina*, *Hevea brasiliensis*; t3, *Betula verrucosa*.

**Figure 2:**
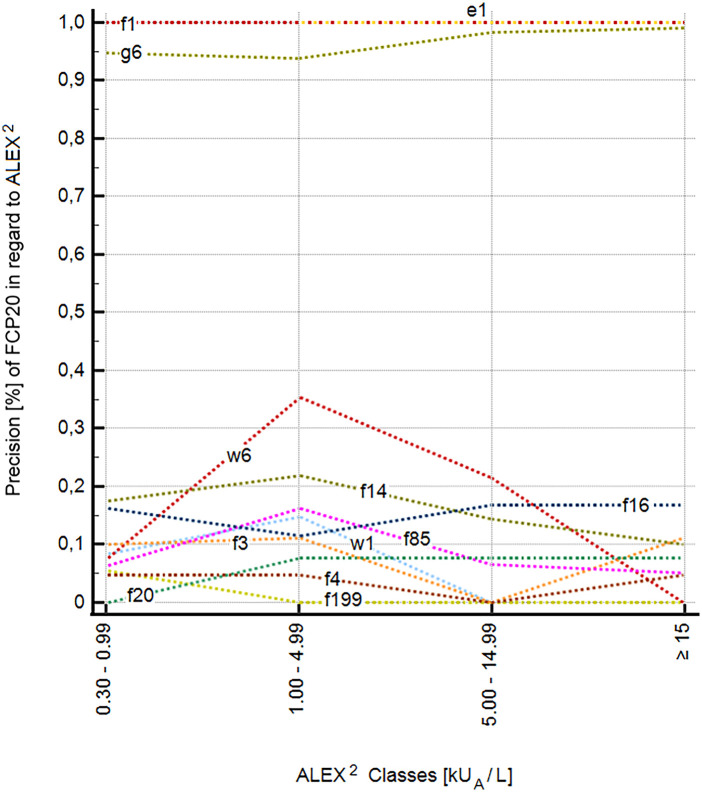
No increase in precision of 12 individual allergens of the FCP20 with increasing specific IgE values to extracts and components in ALEX^2^. e1, *Felis domesticus*; f1, *Ovum gallinae*; f3, *Gadus morhua*; f4, *Triticum aestivum*; f14, *Glycine max*; f16, *Juglans regia*; f20, *Prunus dulcis*; f85, *Apium graveolens*; f199, *Bos domesticus*; g6, *Phleum pratense*; w1, *Ambrosia artemisiifolia*; w6, *Artemisia vulgaris*.

A total of eight allergens or allergen mixtures in the FCP20 test, namely, *Ficus benjamina*, *Hevea brasiliensis* (kx1); the inhalant allergens, *Anthoxanthum odoratum*, *Dactylis glomerata*, *Lolium perenne*, *Poa pratensis* (gx17), *Secale cereale* (g12), *Betula verrucosa* (t3), *Canis familiaris* (e5), *Dermatophagoides farinae*, and *Dermatophagoides pteronyssinus* (dx2); and the food allergens, *Arachis hypogaea* (f13) and *Corylus avellana* (f17), showed an increase in precision with increasing sensitization levels in ALEX^2^ (Figure 1). This suggests that higher levels of sensitization in ALEX^2^ result in improved performance of FCP20. Twelve allergens, namely, the inhalant allergens *Ambrosia artemisiifolia* (w1), *Artemisia vulgaris* (w6), *Phleum pratense* (g6), *Felis domesticus* (e1), and the food allergens *Ovum gallinae* (f1), *Bos primigenius taurus* (f199), *Gadus morhua* (f3), *Triticum aestivum* (f4), *Glycine max* (f14), *Apium graveolens* (f85), *Prunus amygdalus* (f20), and *Juglans regia* (f16), showed no significant increase in precision with increasing sensitization level (Figure 2).

The concordance correlation coefficient (CCC) and the intraclass correlation coefficient (ICC) for all FCP20 allergens and the corresponding components and extracts in ALEX^2^ were 0.40 (95% CI: 0.37–0.42) and 0.58 (95% CI: 0.55–0.60), respectively.

The sensitivity of the FCP20 improves when exclusively test subjects with clinical symptoms were considered. Sensitivity increases for both inhalation and food allergens in patients with bronchial asthma, current eczema, or severe allergic symptoms with a slightly decreased specificity (Table 4).

**Table 4:** Stratification of diagnostic accuracy and predictive values according to clinical symptoms.

FCP20 allergens	Participants with current bronchial asthma (*N* = 58)	Participants with current eczema (*N* = 56)	Participants with severe allergic symptoms (*N* = 86)
A. FCP20 inhalant allergens			
Sensitivity (%)	84.9 (78.2–90.2)	87.3 (80.2–92.6)	83.7 (77.3–88.9)
Specificity (%)	72.0 (67.5–76.2)	70.0 (80.2–92.6)	70.7 (67.2–74.1)
PPV (%)	51.8 (47.7–55.9)	45.8 (42.0–49.8)	41.6 (38.4–44.9)
NPV (%)	93.1 (90.2–95.2)	95.0 (92.3–96.8)	94.6 (92.5–96.1)
B. FCP20 food allergens			
Sensitivity (%)	42.5 (32.0–53.6)	41.8 (31.5–52.6)	47.5 (38.4–56.8)
Specificity (%)	89.6 (86.5–92.2)	87.9 (84.6–90.7)	89.8 (87.4–91.9)
PPV (%)	42.1 (33.7–50.9)	40.0 (32.1–48.5)	43.3 (36.5–50.3)
NPV (%)	89.8 (88.0–91.3)	88.7 (86.8–90.3)	91.3 (89.8–92.5)
C. FCP20 total allergens			
Sensitivity (%)	69.5 (63.2–75.2)	68.2 (61.6–74.3)	68.7 (63.1–74.0)
Specificity (%)	81.4 (78.7–83.9)	79.3 (76.5–81.9)	80.6 (78.5–82.6)
PPV^†^ (%)	49.6 (45.3–53.2)	44.2 (40.4–48.1)	42.1 (38.9–45.3)
NPV^‡^ (%)	91.1 (89.4–92.6)	91.2 (89.5–92.7)	92.6 (91.4–93.7)

*N*, number of patients; PPV, positive predictive value; NPV, negative predictive value. The 95% CI is shown in brackets.

## 4 Discussion

The evaluation of the FCP20 test resulted in an overall sensitivity of 43.3% (95% CI: 40.3%–46.2%) and a specificity of 92.1% (95% CI: 91.1%–93.0%) in comparison with the multiplex test ALEX^2^ used in our study. This is in contrast to the study by Rübenhagen et al. (14), which showed a much higher overall sensitivity of the FCP20 test (76%) compared with the ImmunoCAP and a lower overall specificity of 80%.

One difficulty in assessing the overall sensitivity and specificity of the FCP20 relates to the different prevalences of sensitizations between 0.9% and 80.5% for individual allergens as determined by ALEX^2^, since a change in prevalence from very low values to high values can change the sensitivity and specificity up to 40% (8, 18). Furthermore, our study did not compare FCP20 allergens with single components of the ImmunoCAP, as utilized by Rübenhagen et al. (14), but used all comparable components and extracts in ALEX^2^. Specific IgE against allergens with a low prevalence can lead to a positive result in the ALEX^2^ and may not be detected in the FCP20 as they are underrepresented. This reduces overall sensitivity and could explain the discrepancy in overall sensitivity of the FCP20 with the previously published study by Rübenhagen et al. (14).

The observed overall superior sensitivity [46.3% (95% CI: 43.0%–49.6%)] and specificity [95.6% (95% CI: 94.3%–96.6%)] for inhalant allergens as compared with food allergens [32% (95% CI: 26.1%–38.2%) and 89.7% (95% CI: 88.3%–91.1%), respectively] in the FCP20 have also been observed by Rübenhagen et al. (14).

There are also clear differences within the inhalant allergen and food allergen groups in sensitivity and specificity for the single allergens. For inhalant allergens *Phleum pratense* (g6), a mixture of *Anthoxanthum odoratum*, *Dactylis glomerata*, *Lolium perenne*, and *Poa pratensis* (gx17), showed the highest sensitivity and specificity, while *Ambrosia artemisiifolia* (w6) exhibited the lowest sensitivity and specificity. We hypothesize that the low specificity of *Ambrosia artemisiifolia* (w6) [62.8% (95% CI: 56.7%–68.6%)], among inhalation allergens, and of *Apium graveolens* (f85) [67.2% (95% CI: 59.7%–74.2%)], among food allergens, is a result of co-sensitizations with allergens of the same allergen families or presence of cross-reacting CCDs.

Babakhin et al. (12) reported a high sensitivity for house dust mite (88.2%) and a high specificity for dog dander (100%) in the FCP20 compared with ImmunoCAP. We found an overall sensitivity for house dust mite of 19.7% (95% CI: 10.9%–31.3%), for the individual components Der f 1 33.3% (95% CI: 17.3%–52.8%), Der f 2 20.5% (95% CI: 9.8%–35.3%), Der p 1 33.3% (95% CI: 18.0%–51.8%), Der p 2 20.0% (95% CI: 14.7%–94.7%), Der p 5 46.2% (95% CI: 19.2%–74.9%), Der p 7 50.0% (95% CI: 18.7%–81.3%), Der p 10 60.0% (95% CI: 14.7%–94.7%), Der p 11 0% (95% CI: 0%–21.8%), Der p 20 16.7% (95% CI: 0.4%–64.1%), and Der p 21 75.0% (95% CI: 34.9%–96.8%). For dog epithelia, the overall specificity was 98.1% (95% CI: 94.5%–99.6%).

Only the perennial inhalant allergens *Dermatophagoides pteronyssinus*, *Dermatophagoides farinae*, *Aspergillus fumigatus*, and *Alternaria alternata* and dog dander were tested in these patients. For *Alternaria alternata*, *Aspergillus fumigatus*, and dog dander, data were available for only 16, 15, and 9 patients, respectively. All patients tested negative for the first three allergens by ImmunoCAP, whereas all 17 patients tested positive for *Dermatophagoides pteronyssinus* and *Dermatophagoides farinae* in ImmunoCAP (12). *Alternaria alternata* and *Aspergillus fumigatus* are only present in the Asia panel of the FCP20 and not in the Atopy panel of the FCP20 that we used for our study.

One weakness of the study of Babakhin et al. (12) is that sensitivity was calculated only for house dust mites, where the ImmunoCAP detected positive sensitizations in all 17 sera. Therefore, the authors could not calculate the specificity for house dust mites on the one hand. On the other hand, they calculated the specificity for *Aspergillus fumigatus*, *Alternaria alternata*, and dog dander without a single positive sensitization in the ImmunoCAP. The reported sensitivity and specificity are not from the same allergen, which is misleading, and therefore, those test results should be treated with caution.

Kamath et al. (13) studied 14 Australian allergic patients with the FCP20 and compared it with the results of the ImmunoCAP. They reported a sensitivity, specificity, and accuracy of 91.8%, 80.2% and 81.7%, respectively. This high sensitivity is in contrast to our overall sensitivity of only 43.3% (95% CI: 40.3%–46.2%) and could again be caused by the lower prevalence of sensitizations in our study. On the other hand, the specificity of 92.1% (95% CI: 91.1%–93.0%) found in our patients is higher, while the accuracy of 79.2% in our patients is similar. Kamath et al. did not specify which data they used to calculate sensitivity, specificity, and accuracy. Defining the FCP20 level 1 as a lack of sensitization, a calculation of their data yielded a sensitivity of 45.3%, a specificity of 83.9%, and an accuracy of 76.5%. Kamath et al. also presented data from 12 Australian subjects and 59 European subjects who were tested for hazelnut and were all classified as class 0 with ImmunoCAP. Five of the Australian patients and 18 of the European patients were classified as at least level 2 with FCP20. The authors described that these discrepancies may be due to non-specific binding to carbohydrate moieties.

If we consider the FCP20 test performance as a function of clinical symptoms, the overall sensitivity of the FCP20 in patients with bronchial asthma increased to 69.5%, and the sensitivity for inhalation allergens rose to 84.9%. However, the sensitivity for food allergens increased only slightly. A similar result was obtained when patients with either eczematous skin problems or severe allergic symptoms were evaluated. For the latter, the sensitivity for food allergens of the FCP20 increased to 47.5%. For this reason, the use of the FCP20 is better suited in a selected patient population compared with an unselected patient population.

It is also important to emphasize that, with increasing levels of sensitization in the ALEX^2^, not all allergens showed an increase in positivity in the FCP20. This may be related to the different allergens or the different binding capacity of sIgE in both test platforms. An increase in the sensitivity of the FCP20 with an increase in the level of sIgE in ALEX^2^ was found for the inhalation allergens *Anthoxanthum odoratum*, *Dactylis glomerata*, *Lolium perenne*, *Poa pratensis* (gx17), *Secale cereale* (g12), *Betula verrucosa*, *Ficus benjamina*, *Hevea brasiliensis* (kx1), *Canis familiaris* (e5), *Dermatophagoides farinae*, and *Dermatophagoides pteronyssinus* (dx2) and the food allergens, *Arachis hypogaea* (f13) and *Corylus avellana* (f17).

When prevalence is taken into account, inhalation allergens, especially grass pollen [*Phleum pratense* (g6), *Secale cereale* (g12), *Anthoxanthum odoratum*, *Dactylis glomerata*, *Lolium perenne*, *Poa pratensis* (gx17)], performed better than the majority of food allergens [except for *Juglans regia* (f16)].

The low sensitivity (43.3%) of FCP20 limits its effectiveness as a general screening tool, as it may fail to detect sensitizations, resulting in potential false negatives. Therefore, it should be considered a supplementary tool, rather than a stand-alone diagnostic tool: The FCP20 test may miss true sensitizations, particularly in patients with mild or atypical symptoms, resulting in delayed diagnoses or inappropriate disease management. FCP20 is also less reliable for screening populations with low allergen prevalence or unclear clinical histories. Its false-negative rate could lead to misdiagnoses. However, the test shows improved sensitivity in patients with severe allergic symptoms (e.g., asthma or eczema). In primary care, the test should be used selectively and always in conjunction with clinical evaluation and additional diagnostic methods (e.g., skin prick tests) to avoid misinterpretation.

Our cohort consists of individuals with allergic symptoms or a known history of allergies. Recruitment did not take place through an allergy center; rather, it reflects the situation at a primary care center. Nevertheless, it can be assumed that there is a minimal selection bias due to the small negative control group.

We are aware that a comparison of extracts with components has some limitations. Furthermore, measurements of specific IgE should ideally be compared with the ImmunoCAP, as a reference method (19, 20). Although a recent study showed a slightly lower sensitivity for seasonal allergens of ALEX^2^ in comparison with ImmunoCAP (21), we chose ALEX^2^ as the reference method in this study since not all extracts and components of the ALEX^2^ test are available for the ImmunoCAP (e.g., Amb a 4, Api g 2, Api g 6, Ara h 15, Cor a 11, Der f 1, Der f 2, Der p 5, Der p 7, Der p 11, Der p 20, Der p 21, Gad m 1, Gly m 8, Jug r 2, Jug r 4, Jug r 6, and Tri a aA_TI). Moreover, the extracts *Bos primigenius taurus* (f199) and *Juglans regia* (f16) of FCP20 are not available for ImmunoCAP. In addition, CCDs can lead to false-positive results with the ImmunoCAP (21, 22), whereas ALEX^2^ includes an inhibitor for CCDs, which reduces 88.5% of CCD-positive signals detected by the ImmunoCAP ISAC (17). Nonetheless, in a clinical setting, the correlation between negative or positive results of the FCP20 compared with the ALEX^2^ is of importance. In our study, the ALEX^2^ test was used for comparison, whereby we compared the sum of all components and extracts in the ALEX^2^ test with the FCP20 extracts. Whether the positive signals in ALEX^2^ are reflected by extracts, individual components, or several components is of secondary importance. Consequently, our findings on sensitivity and specificity diverge from previously published studies that compared the FCP20 with the singleplex ImmunoCAP, in which only individual components or extracts were analyzed. A positive match was defined as a positive FCP20 test result with at least one corresponding positive result for an extract or a component in ALEX^2^. Caution should be exercised when making comparisons with previous studies using singleplex ImmunoCAP, as these studies only evaluated one ImmunoCAP extract or component against a single FCP20 extract. Anna Ringauf, one of the authors who performed the ALEX^2^ test, is an employee of MADx, which could introduce a potential bias. However, she did not participate in data analysis.

In conclusion, the FCP20 demonstrates a high specificity of 92.1% (95% CI: 91.1%–93.0%) and may be considered for the exclusion of sensitization to certain allergens, but its low sensitivity of 43.3% (95% CI: 40.3%–46.2%) limits its usefulness as a general screening tool. This general statement does not apply to individual extracts such as grass pollen, as they are easily detected using the FCP20.

## Data Availability

The raw data supporting the conclusions of this article will be made available by the authors, without undue reservation.
