# Supplementary figures and images for: Allergy diagnostic performance of FastCheckPOC 20 Atopy
Source: Front Allergy. 2025 Oct 24;6:1669268. doi: 10.3389/falgy.2025.1669268 (PMC12592802; doi:10.3389/falgy.2025.1669268)

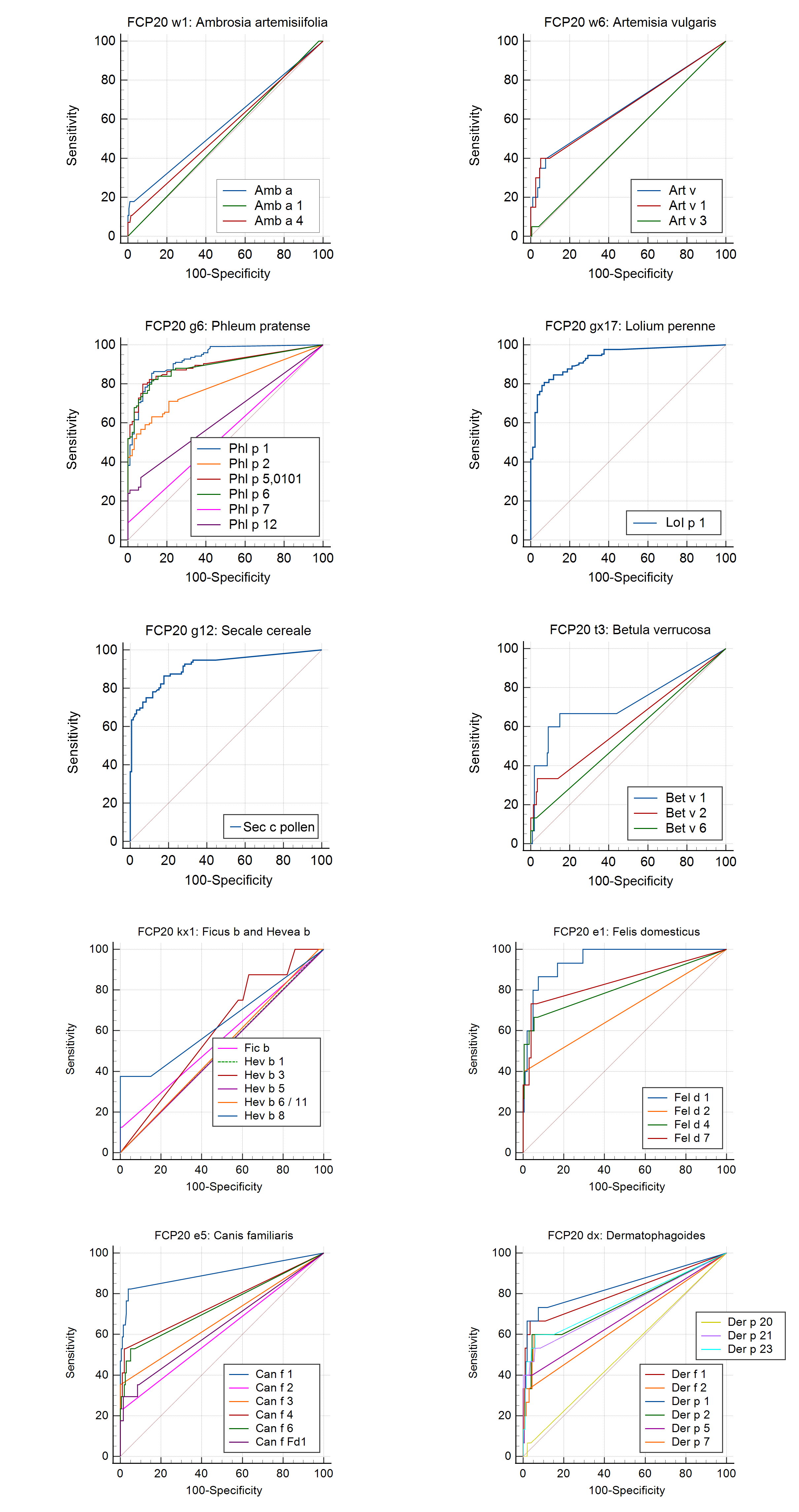

Supplement: Supplementary file 1 [file Image1.jpeg]

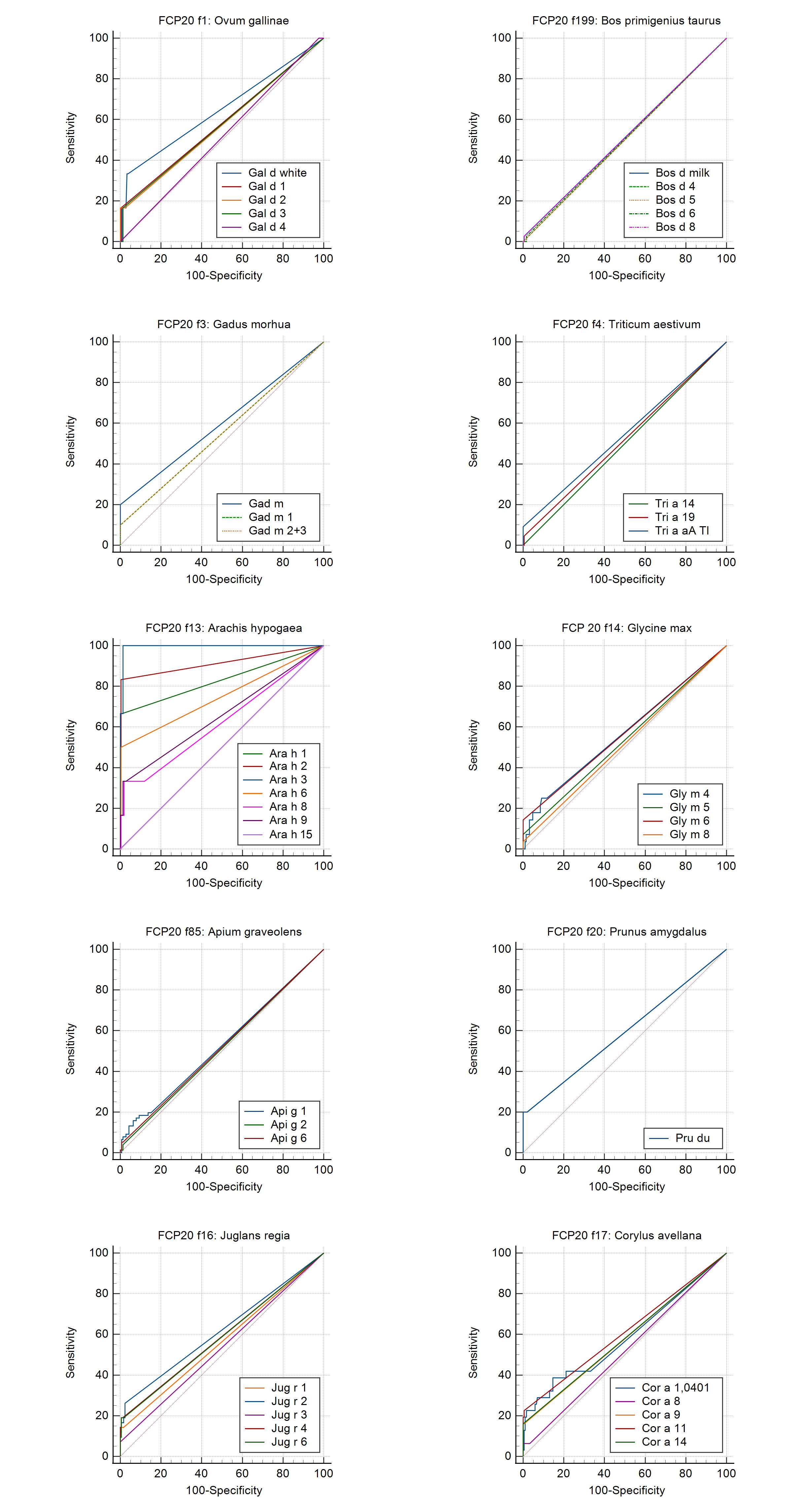

Supplement: Supplementary file 2 [file Image2.jpeg]
